# Evaluation of Current Knowledge, Awareness and Practice of Spirometry among Hospital -based Nigerian Doctors

**DOI:** 10.1186/1471-2466-9-50

**Published:** 2009-12-14

**Authors:** Olufemi O Desalu, Olusegun A Busari, Cajetan C Onyedum, Fatai K Salawu, Olusegun A Obateru, Kelechukwu C Nwogu, Alakija K Salami

**Affiliations:** 1 Department of Medicine, University of Ilorin Teaching Hospital Ilorin, Nigeria; 2 Department of Medicine, Federal Medical Centre Ido-Ekiti, Nigeria; 3 Department of Medicine, University of Nigeria Teaching Hospital Enugu, Nigeria; 4 Department of Medicine, Federal Medical Centre, Yola, Nigeria; 5 Department of Medicine, Federal Medical Centre Birnin Kebbi, Nigeria

## Abstract

**Background:**

Spirometry is a cost-effective diagnostic tool for evaluation of lung function and for case-finding in a resource-limited setting. The acceptance of this test depends on the awareness of its indications and the ability to interpret the results. No studies have assessed the knowledge of spirometry among Nigerian doctors. The aim of this study was to evaluate the current knowledge, awareness and practice of spirometry among hospital-based Nigerian doctors.

**Methods:**

We carried out a cross-sectional survey among 321 doctors working in Nigerian hospitals between March 2008 and June 2008. Information on knowledge, awareness, practice of and barriers to spirometry were obtained using a pre-tested, self-administered structured questionnaire and the data were then analysed.

**Results:**

Of the 321 doctors that participated, 108 (33.6%) reported that they have good knowledge of spirometry. One hundred and ninety-five (60.7%) were aware of the importance of spirometry in aiding the diagnosis of respiratory diseases; 213(66.4%) were aware of the importance of spirometry in determining the severity of diseases. Medical school was the most common source of knowledge on spirometry (64.5%). Eighty-one (25.2%) doctors reported having a spirometer in their hospitals. Doctors having access to a spirometer used it more frequently for aiding the diagnosis of COPD (40.7% vs.27.5%) and for monitoring of asthma (18.5% vs.11.3%) than those without access to a spirometer. The doctors working in University Teaching Hospitals and Federal Medical Centres (FMC) (22.4% vs. 4.5%) and those having access to a spirometer (40.7 vs.11.3%) were very confident of interpreting spirometry results compared to those working in District and General Hospitals and without access to a spirometer. Irrespective of access to a spirometer or the type of hospital they were employed in, doctors reported that unavailability of a spirometer was the greatest barrier to its use (62.5%) followed by lack of awareness about its usefulness (17.2%).

**Conclusion:**

The knowledge and practice of spirometry were poor among hospital-based Nigerian doctors because of unavailability of spirometers in most hospitals. These findings have implications for further evaluation, planning and management of patient care in respiratory disease. Spirometers should be made available in all hospitals, and the knowledge of spirometry should be improved among doctors.

## Background

Spirometry is the timed measurement of dynamic lung volumes during forced expiration and inspiration to quantify how effectively and how quickly the lungs can be emptied and filled [1]. Spirometry is often used to improve diagnosis and to monitor acute and chronic respiratory diseases. Respiratory diseases refer to a variety of preventable and non-preventable chronic diseases of the airways and other structures of the lung [2]. Research has shown that many respiratory illnesses can be prevented, diagnosed at an early stage and controlled with adequate management [3]. Appropriate management of a respiratory morbidity requires obtaining a good history of symptoms and clinical signs as well conducting relevant investigations. In most low-income countries, imaging and endoscopy services are mostly unavailable, and doctors have limited access to tools like chest radiographs and spirometry for evaluation of respiratory diseases. Spirometry is essential for the identification of many respiratory disease states and for the objective monitoring of responses to therapy [4,5]. A spirometry test substantially improves diagnostic competence and case-finding of diseases like COPD if applied in a pre-selected high-risk population [6]. A spirometry test can facilitate diagnosis and aid in the management of most restrictive lung diseases. Abnormal spirometry is an indicator of increased risk for premature death from all causes, and several insurance companies' recommends indicate it as a prerequisite for obtaining a life insurance policy [5]. Spirometry can also be used preoperatively to determine the cardio-respiratory status of surgical patients, to measure lung age and to aid in smoking cessation [5]. Although studies have been published on spirometry reference values and ventilatory function tests in chronic obstructive lung disease among the Nigerian population [7,8], there are no studies that evaluate the knowledge, awareness and practice of spirometry among doctors in Nigeria. The aim of this study was to evaluate the current knowledge, awareness and practice of spirometry among hospital-based Nigerian doctors.

## Methods

### Study setting and Design

This is a cross-sectional study involving 321 doctors working in Nigerian hospitals between March 2008 and June 2008. Nigeria is located in West Africa and is divided into 6 geopolitical zones. The country has a population of 145 million, with an annual growth rate of 2.2% [9]. The ratio of district and private hospitals to the University Teaching Hospitals (UTH) and Federal Medical Centres (FMC) is 10:1.

### Sample selection

This survey was conducted in five of the six geopolitical zones which can be considered a representative sample of Nigerian doctors. The investigators selected one district hospital, one privately owned hospital and one University Teaching Hospital or Federal Medical Centre (FMC) in each geopolitical zone. These hospitals were selected for reasons of convenience and easy coordination by the zonal investigators. (See appendix for the list of selected hospitals.) A total of 15 Nigerian hospitals (5 geopolitical zones × 3) were selected for the study. The zonal investigators coordinated the administration of the questionnaires. The doctors in the participating hospitals were selected by simple random sampling to minimize bias; however, interns and laboratory physicians were excluded from this study.

### Survey instrument

We used a pre-tested, self-administered structured questionnaire that was prepared using statements on spirometry by the American Thoracic Society (ATS) and the European Respiratory Society [4], and questions that have been used in two previous studies [10,11]. The questionnaire was in English, which is the official language of communication in Nigeria. For construction and content validity, the questionnaire was reviewed by two respiratory physicians. There was 80% agreement on the 25 questions and their wording. We tested the questionnaire for face validity in a pilot study on 20 doctors to ascertain if the questions were acceptable and their wording was well understood by the respondents.

The test-retest reliability of the instrument at a two-week interval was r = 0.78 and the internal consistency was Crohnbach's alpha = 0.71. (See the additional file 1 for the questionnaire.)

The selected doctors gave oral consent to the zonal investigators or signed an informed consent form to participate in the study. A few doctors, however, preferred oral consent over signing the informed consent form, in order to avoid personal identification. The purpose of the study was explained to the doctors in the information note attached to questionnaire and their right to withdraw was ensured. Physicians who consented to participate in the study were requested to complete the questionnaire according to their own knowledge and awareness of the subject, in a quiet environment without interference from their colleagues. Socio-demographic information of each participating physician and data regarding location of practice, years of experience, number of patients with respiratory diseases seen per week, and tobacco smokers encountered per week were obtained. In addition, each doctor was asked to respond to 25 questions: 8 were related to awareness, self-reported knowledge and interpretation of spirometry; 11 pertained to spirometry practice in their hospitals, 6 were related to attendance of general CME programmes, sources of current knowledge, availability of spirometers in hospitals and barriers to use of spirometry. The responses to the questionnaires were collated by the zonal investigators and sent to the statistician. Anonymity and confidentiality of the participants were guaranteed.

### Data analysis

Data obtained were analysed using SPSS statistical software version 15 (SPSS Inc., Chicago, IL, USA). Frequency and descriptive statistics were used to examine the general characteristics of the physicians. The participating doctors were stratified into two groups on the basis of the reported availability of a spirometer in their hospital and type of hospital where they were employed (University Teaching Hospital/FMC or district/private Hospital). The responses of the doctors to questions on practice of and barriers to spirometry were analysed according to the stratification. Chi square test was used to assess the significance of the responses and a P value of < 0.05 was considered statistically significant.

### Ethical approval

The study was approved by the ethics and research committee of the Federal Medical Centre of Ido-Ekiti, Nigeria.

## Results

Of the 450 questionnaires that were distributed, 321 were returned by the doctors, leading to a response rate of 71.3%. Two hundred fifty-two (78.5%) of the 321 doctors were men and 69 (21.5%) were women; their mean age was 34 ± 4.4 years. Eighty-one (25.2%) doctors reported having a spirometer in their hospitals. Other characteristics of the participating doctors are given in table 1.

**Table 1:** Characteristics of the participating doctors

Characteristics	Mean (SD), n (%)
**Age**	34(4.4)yrs
**Respiratory diseases cases/wk**	12.7(12.4)
**Sex**	
Male	252(78.5)
Female	69(21.5)
**Years of practice**	
<5	169(52.6)
>5	152(47.4)
**location of practice**	
Urban	267(83.2)
Rural	54(16.8)
**Types of hospital**	
District/private	66(20.6)
University/FMC	255(79.4)
**Availability of spirometer in workplace**	
Yes	81(25.2)
No	240(74.8)
**Awareness of guideline on spirometry interpretation**	
Yes	90(28.0)
No	231(72.0)

SD = standard deviation

### Awareness of the role of spirometry

Of 321 the medical doctors that participated in this study, 195 (60.7%) reported that spirometry was very important in the diagnosis of respiratory diseases; 213(66.4%) reported that it was very important in determining the severity of the respiratory disease. Furthermore, the majority of the doctors, 228 (71.0%), stated that spirometry was useful for monitoring the progression of a respiratory disease, 180 doctors (56.1%) reported that the spirometry was useful for surveillance of occupational lung disease and 171(53.3%) reported the test to be useful for pre-operative evaluation of surgical patients and assessment of individuals applying for a life insurance policy. In this study, 135 doctors (42.1%) reported lack of awareness regarding spirometry role in determining the prognosis of a respiratory disease.

### Knowledge of spirometry

In response to questions determining the lung function tests that would be performed on patients with suspected bronchial asthma or COPD, 189 (58.9%) of the surveyed doctors reported that they would measure peak flow rate, 63 (19.6%) stated that they would opt for baseline spirometry and 39 (12.1%) said that they would perform acute reversibility testing using a bronchodilator. One hundred fifty-six (48.6%) doctors reported that they have fair knowledge of spirometry, 108 (33.6%) reported to have good knowledge while 57 (17.8%) stated that they have poor knowledge of spirometry. The majority of the participants, 207 (64.5%), reported that medical school was the most common source of knowledge on spirometry, followed by continuing medical education programmes 87 (27.1%), internet websites 15 (4.7%) and medical textbooks and journals 12 (3.7%). This study also revealed that 69(21.5%) of the surveyed doctors had attended a CME programme in general medicine in the previous year, while 21(6.5%) had done so in the previous 2 to 5 years.

### Practice of spirometry

We found that 53 (65.4%) of the 81 doctors with access to a spirometer used spirometry more frequently as compared to 46 (19.2%) of 240 doctors without access. Doctors with access to a spirometer frequently used it for aiding the diagnosis of COPD (40.7% vs.27.5%), monitoring of asthma (18.5% vs.11.3%), assessment of pre-employment baseline lung function (11.1% vs.11.0%) compared to those without access to it. Sixty of the 321 participating doctors (18.7%) reported that they were very confident of interpreting spirometry results, 168 (52.3%) were slightly confident and 93 (29%) were not confident of their interpretation skills. Thirty three (40.7%) of the doctors with access to spirometers reported that they were very confident of interpreting spirometry results compared to 11.3% of those without access to spirometers. Other responses to questions on the practice of spirometry are given in table 2. Further stratification of the responses of doctors according to the type of hospital showed that doctors working in University Teaching Hospital and Federal Medical Centre reported that they frequently request spirometry for diagnosing COPD (34.1% vs.18.2%), pre-employment evaluation (10.6% vs.9.1%) and were very confident of interpreting spirometry results (22.4% vs.4.5%) compared to doctors working in district and private hospitals. Relevant data are given in table 3.

**Table 2:** Distribution of doctors' responses to questions on practice of spirometry as related to access to spirometry in the hospitals

	Access to spirometry		
	Yes	No	
Questions related to practice of spirometry	n (%)	n (%)	Total
1. How frequently do you use spirometry in your practice?			
Very often	53(65.4)	46(19.2)	99(30.8)
Occasionally	25(30.9)	128(53.3)	153(47.7)
Rarely	0(0.0)	54(22.5)	54(16.8)
Never/can't recall	3(3.7)	12(5.0)	15(4.6)
2. How frequently do you request spirometry for monitoring for asthma?			
Frequently depending on control	15(18.5)	27(11.3)	42(13.1)
Once a year	9(11.1)	9(3.8)	18(5.6)
Every follow up	39(48.2)	48(20.0)	87(27.1)
Never	18(22.2)	156(65.0)	174(54.2)
3. How frequently do you request spirometry for diagnosing COPD?			
Occasionally	24(29.6)	30(12.5)	54(16.8)
Very frequent	33(40.7)	66(27.5)	99(30.8)
Rarely	6(7.4)	30(12.5)	36(11.2)
Never	18(22.2)	114(47.5)	132(41.1)
4. How frequently do you request for spirometry pre employment test			
Very frequent	9(11.1)	24(10.0)	33(10.3)
Occasionally	12(14.8)	30(12.5)	42(13.1)
Rarely	51(63.0)	138(57.5)	189(58.9)
Never/can't recall	9(11.1)	48(20.0)	57(17.8)
5 How confident are you at interpreting spirometry			
Not confident	12(14.8)	75(31.3)	87(27.1)
Slightly confident	30(37.0)	138(57.5)	168(52.3)
Very confident	33(40.7)	27(11.3)	60(18.7)
Don't know	6(7.4)	0(0.0)	6(1.9)

P < 0.001 for responses(1-5)

**Table 3:** Distribution of doctors' responses to questions on practice of spirometry as related to the type of hospitals

	Type of hospital		
	UTH/FMC	DGH/PH	
Questions related to practice of spirometry	n (%)	n (%)	Total
1. How frequently do you use spirometry in your practice?*			
Very often	78(30.6)	21(31.8)	99(30.8)
Occasionally	123(48.2)	30(45.5)	153(47.7)
Rarely	45(17.7)	9(13.6)	54(16.8)
Never/can't recall	9(3.5)	6(9.1)	15(4.6)
2. How frequently do you request spirometry for monitoring?			
for asthma? ^†^			
Frequently depending on control	33(12.9)	9(13.6)	42(13.1)
Once a year	12(4.7)	6(9.1)	18(5.6)
Every follow up	72(28.2)	15(22.7)	87(27.1)
Never	138(54.1)	36(54.6)	174(54.2)
3. How frequently do you request spirometry for diagnosing?			
COPD? ^§^			
Very frequent	87(34.1)	12(18.2)	99(30.8)
Occasionally	39(15.3)	15(22.7)	54(16.8)
Rarely	27(10.6)	9(13.6)	36(11.2)
Never	102(40.0)	30(45.5)	132(41.1)
4. How frequently do you request for spirometry			
pre employment test? ^a^			
Very frequent	27(10.6)	6(9.1)	33(10.3)
Occasionally	30(11.8)	12(18.2)	42(13.1)
Rarely	150(58.8)	39(59.1)	189(58.9)
Never/can't recall	48(18.8)	9(13.6)	57(17.8)
5. How confident are you at interpreting spirometry? ^d^			
Not confident	53(20.8)	24(36.4)	87(27.1)
Slightly confident	129(50.6)	39(59.1)	168(52.3)
Very confident	57(22.4)	3(4.5)	60(18.7)
Don't know	26(10.2)	0(0.0)	6(1.9)

*X ^2 ^= 6.9 df = 3 P =< 0.07, ^†^, X^2 ^= 2.4 df = 3 p = 0.49, ^§ ^X^2 ^= 9.8 df = 3 p = 0.02, ^a ^X^2 ^= 6.5 df = 3

p = 0.09 δ X^2 ^= 121 df = 3 p = 0.02

UTH/FMC = University Teaching Hospital/Federal Medical Centre

DGH/PH = District & General hospital/private Hospital

### Barriers to spirometry practice

**Figure 1:**
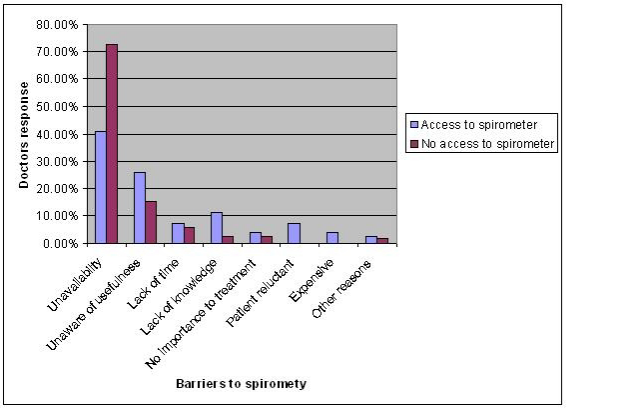
**Barriers to spirometry practice among doctor**.

Irrespective of the doctors' access to a spirometer or the type of hospital they worked in, unavailability, 207 (62.5%) was the greatest barrier to the use of spirometry followed by lack of awareness of its usefulness, 57(17.2%). Other reported barriers are given in table 4 and illustrated in figure 1.

**Table 4:** Self -reported barriers to spirometry practice among doctor as related to the type of hospital (there may be more than one response for the same respondent).

Reasons	TH & FMC	DGH & PH	Total
	**(n = 260)**	**(n = 71)**	**(n = 331)**
			
Unavailabilty	162(62.3)	45(63.3)	207(62.5)
Unaware of usefulness	39(15.0)	18((25.4)	57(17.2)
Lack of time	28(10.8)	0(0.0)	28(8.5)
Lack of knowledge	15(5.8)	0(0.0)	15(4.5)
No impt to treatment	6(2.3)	3(4.2)	9(2.7)
Patient reluctant	6(2.3)	0(0.0)	6(1.8)
Expensive	3(1.2)	0(0.0)	3(0.9)
Other reasons	1(0.4)	5(7.0)	6(1.8)

X^2 ^= 9.79 df = 7 p = 0.201

## Discussion

This study shows that the majority of Nigerian doctors were aware of the role of spirometry in the diagnosis, assessment and progression monitoring of respiratory diseases. However, only 33.6% appreciated the prognostic significance of the spirometry test. Furthermore, majority of the doctors were also aware of its role in the surveillance of occupational lung disease among those at risk of developing work-related diseases. They also acknowledged its role in pre-operative evaluation of surgical patients and baseline evaluation of the lung function among those seeking a life insurance policy. Our data also revealed that few doctors reported having good knowledge of spirometry and the ability to interpret spirometry and this result was in agreement with that of the other studies [10-12]. This finding may be because of lack of emphasis on spirometry in medical schools and in various CME programmes organised by the post-graduate colleges and medical associations. Although the most common source of knowledge on spirometry was medical schools, spirometry was poorly taught in most medical schools in Nigeria as well as in the USA [5]. Further attendance was poor at conference workshops organised by the National Respiratory Society. This trend was reflected in the low attendance figures at CME programmes during the past five years reported in this study. Factors associated with poor attendance at spirometry workshops must be addressed in order to increase the use of spirometry [13]. Kaminsky and colleagues have reported improvement in knowledge and use of spirometry through workshops and seminars [11]. In this study, less than a third (30.8%) of those surveyed frequently used spirometry in their practice. The low utilization and poor practice of spirometry among doctors in this study was similar to reports of other researchers [11-17]. The low utilization of spirometry in our study may be due to several factors. The most common barrier to the practice of spirometry reported by the doctors was unavailability of spirometers in hospitals (64.5%). Surprisingly, even among doctors with access to spirometers, unavailability was reported as the greatest barrier to the use of spirometry. Other investigators have reported uncertainty about the impact of a spirometry test, physician and staff unfamiliarity [11], lack of access to a well-maintained spirometer and expertise [14], logistical limitations preventing patients from accessing lung function laboratories [17] and lack of knowledge [18]. We are not surprised that unavailability was the greatest barrier in this study, as only 25.2% of doctors reported having a spirometer in their current hospitals, as opposed to 66.0% in the USA, 64.2% in Australia and 90.9% in Spain [11,14,15]. Procurement of spirometers is not given top priority by most health administrators because it does not generate profits like other routine investigations. It is ironic that despite the strong epidemiological evidence demonstrating an association between reduced lung function and important clinical outcomes as well as the role of spirometry in early detection of some respiratory diseases [5,19-21]; it lacks acceptability in clinical practice. The spirometer is yet to find its rightful place alongside other routinely used instruments like the glucometer, sphygmomanometer and the electrocardiograph (ECG). The first step towards increasing the acceptance of spirometry is providing simple portable spirometers to the hospitals. Secondly, education on spirometry should be enhanced among doctors as only 18.7% of the surveyed doctors were very confident at interpreting spirometry tests. Our findings have a significant implication for management of respiratory diseases, as mortality from chronic respiratory disease is projected to rise over the next 10 years in Nigeria [22].

### Strengths and limitations

This is an explorative study in Nigeria and sub-Saharan Africa that evaluated the knowledge, awareness and practice of spirometry among doctors. The results of our study will assist clinicians and hospital administrators in ensuring availability of spirometers in their hospitals and in formulating a policy that will enhance the knowledge and practice of spirometry. The limitations of this study were difficulties in obtaining current addresses and employment details of registered practitioners in Nigeria.

## Conclusion

Most Nigerian doctors are aware of the role of spirometry, but they lack adequate knowledge to practice it and interpret the test results in their hospital. Unavailability was the greatest barrier to the use of spirometry. There is a need to make spirometer widely available in hospitals in order to encourage the practice of spirometry among doctors. We also need to address the other barriers obstructing the practice of spirometry and improve the knowledge of spirometry among doctors. These measures will eventually translate into improvements in the quality of respiratory care.

## Competing interests

The authors declare that they have no competing interests.

## Authors' contributions

OOD conceived and designed the study, conducted data collection and analysis and wrote the first draft of the manuscript. OB coordinated and contributed to final draft of the manuscript. CCO contributed to data collection technical and editorial review. FKS assisted in data collection and final drafting. OAB contributed to data collection, or analysis and interpretation of data. KCN contributed to data collection and regional coordination of the study. AKS contributed to editorial review and final draft of the manuscript. All authors read and approved the final manuscript.

## Appendix 1

### List of selected hospital in nigeria that participated in the study

Enugu State University Teaching Hospital Park lane Enugu

University of Nigeria Teaching Hospital Enugu, Nigeria

Federal Medical Centre Abakaliki, Ebonyi State

Mile 4 Missionary Hospital Abakaliki, Ebonyi State

Federal Medical Centre Yola, Adamawa State

Adamawa State Specialist Hospital, Adamawa State

Galbose Hospital Yola, Adamawa State

University of Ilorin Teaching Hospital, Ilorin

Sobi Specialist Hospital, Sobi, Ilorin

Olanrewaju Hospital, Ilorin

Federal Medical Centre Ido-Ekiti, Nigeria

University Teaching Hospital, Ado-Ekiti, Nigeria

Adeniyi hospital Ado-Ekiti, Nigeria

Federal Medical Centre Birnin Kebbi, Nigeria

Sir Yahaya Memorial Specialist Hospital, Birnin-kebbi

Sokoto Clinic, Sokoto 

## Pre-publication history

The pre-publication history for this paper can be accessed here:

http://www.biomedcentral.com/1471-2466/9/50/prepub

## Supplementary Material

Additional file 1 **Survey instrument for the study**. Details on questionnaire items and wording Click here for file
