# Supplementary material for: Evaluation of Current Knowledge, Awareness and Practice of Spirometry among Hospital -based Nigerian Doctors
Source: BMC Pulm Med. 2009 Dec 14;9:50. doi: 10.1186/1471-2466-9-50 (PMC2803443; doi:10.1186/1471-2466-9-50)
Supplement: Additional file 1 — Survey instrument for the study. Details on questionnaire items and wording [file 1471-2466-9-50-S1.DOC]

**Knowledge and practices of spirometry among doctors in Nigeria**

**Study no□□□ (official use only)**

**Please answer the questions by ticking the box after your chosen response**

**A. Demographic data**

Age: □□ Sex: M □ F□

Years of practice: □□

Current position in your hospital:

SHO□ medical officer□ Resident□ others (specify)………..

Which types of hospital are you currently employed?

Teaching □ FMC/Specialist □ District /general □ private□

Where is the location of your hospital? urban area □ rural area □

What is the number of seen in your clinic per day? □□

How many of your patients have respiratory diseases?□□

What is the number of tobacco user seen in your clinic per day? □□

**B knowledge and awareness**

1. How important is spirometry in the diagnosis of respiratory disease?

Not important**□**  fairly **□**  very**□** don’t know **□**

1. How important is spirometry in determining the severity of respiratory disease?

Not important**□**  fairly **□**  very**□** don’t know **□**

1. How important is spirometry in determining the prognosis of respiratory disease?

Not important**□**  fairly **□**  very**□** don’t know **□**

1. How useful is spirometry for monitoring of progression of respiratory disease?

Not useful **□**  fairly useful **□**  very useful **□** don’t know **□**

1. How useful is spirometry in confirming clinical diagnosis based on respiratory? symptoms? Not useful**□**  fairly useful**□**  very useful**□** don’t know **□**
2. How important is spirometry for surveillance of occupational lung disease?

Not important**□**  fairly **□**  very**□** don’t know **□**

1. How important is spirometry for pre-operative evaluation of surgical/ baseline lung evaluation in life insurance policy?

Not important**□**  fairly **□**  very**□** don’t know **□**

1. Which of the following tests would you conduct for suspected asthma or COPD?

Peak flow rate**□** baseline spirometry **□** Reversibility test**□** don’t know**□**

**Practices**

1. How frequently do you use spirometry in your practice?

Very often **□**  occasionally **□** rarely **□** Never /can’t recall**□**

1. How frequently do you request spirometry for monitoring for asthma?

Frequently depending on control**□** once a year**□**

Every visit**□** Never /can’t recall**□**

1. How frequently do you request spirometry for diagnosing COPD?

Very frequent**□** Occasionally**□** Rarely**□** Never/can’t recall**□**

1. How frequently do you request spirometry for evaluation of other lung diseases?

Very frequent**□** Occasionally**□** Rarely**□** Never/can’t recall**□**

1. How soon do you request or use spirometry for evaluation of acute exacerbations of asthma, COPD and allergic cough?

Immediately on presentation **□** Once affordable**□** Never/can’t recall**□**

1. How frequently do you request for spirometry pre employment test?

Very frequent**□** Occasionally**□** Rarely**□** Never/can’t recall**□**

1. How frequently do you request spirometry for monitoring of lung function of spinal cord patients

Only if indicated**□** often**□** Rarely**□** Never/can’t recall**□**

1. How confident are you at interpreting spirometry

Not confident**□** slightly confident**□** Very confident **□** Don’t know**□**

1. How frequently do you request spirometry for monitoring of chronic lung diseases (lung fibrosis, e.t.c)

3-6months**□** Once a year**□** every follow up visit**□** Never /can’t recall**□**

1. What is your current knowledge of Spirometry?

Poor**□**  Fair**□**  Good**□** don’t know**□**

1. What are your sources of knowledge and information on spirometry?

Medical school **□** CME programme (conferences/seminars/update courses) **□**

Textbooks and medical journal**□** websites**□**  others (specify) …………..

1. When last did you attend a CME programme? < 12months **□**  2 -5 years**□**
2. Do you have spirometer in your hospital?

yes**□** no**□**  don’t know**□**

1. Does your hospital plan to procure a spirometer?

yes**□** no**□**  don’t know**□**

1. Are you aware of any guideline on spirometry?

yes**□** no**□**  don’t know**□**

1. Are you aware of GINA or GOLD guideline for the management of asthma or COPD respectively?

yes**□** no**□**  don’t know**□**

1. What are the barrier(s) that are preventing you from using spirometry in your hospital?

Unavailabilty**□** Unaware of usefulness**□** Lack of time**□**

Lack of knowledge**□** Not important to treatment**□** Patient reluctant **□**

Expensive **□** Other reasons…………………………………………

**Thank you for your participation**

**NB: All information obtained in the study will be kept Confidential and**

**used for medical research only**
